# Homozygous Nonsense Variant in the GJA4 Gene Associated With Increased Fetal Nuchal Fold Thickness and Abnormal Fetal Ductus Venosus Termination: A Case Report

**DOI:** 10.7759/cureus.97033

**Published:** 2025-11-17

**Authors:** Binodini Chauhan, Nilamben A Prajapati, Sneha Sagarkar, Pramila Menon, Tushar Kachhadiya, Shalin Vaniawala, Salil Vaniawala, Vasundhara Tamhankar, Parag M Tamhankar

**Affiliations:** 1 Fetal Medicine, Government Medical College, Surat, Surat, IND; 2 Obstetrics and Gynaecology, Government Medical College, Surat, Surat, IND; 3 Central Research Facility, Dr. D. Y. Patil Medical College, Hospital & Research Centre, Pune, IND; 4 Pediatrics, Dr. D. Y. Patil Medical College, Hospital & Research Centre, Pune, IND; 5 Genetics, SN GeneLab, Surat, IND; 6 Genetics, Centre for Medical Genetics, Mumbai, IND; 7 Genetics, SN GeneLabs, Surat, IND

**Keywords:** chromosome microarray, exome sequencing, homozygosity, lymphatic abnormalities, nonsense mutation

## Abstract

The *GJA4* gene encodes connexin 37 or Gap junction protein alpha-4. Gap junction proteins are required for lymphatic valvulogenesis. It is known that homozygous knockout of the Gja4 gene in mice leads to lymphatic system dysfunction and absent venous valves. In this report, we identify for the first time a homozygous nonsense variant in the *GJA4* gene, c.97delC (transcript ID NM_002060.3) or p.Arg33Alafs*98, or chr1:g.34794309delC (GRCh38 format) in a human fetus with increased nuchal fold thickness and abnormal fetal ductus venosus termination. Asymptomatic parents were carriers of the same variant. Additionally, a search of the literature showed that this specific variant in the *GJA4* gene has not been previously documented as a cause of human fetal disease. A STRING (Search Tool for Retrieval of Interacting Genes/Proteins) database analysis showed close interactions between the *GJA4* gene and other genes involved in the causation of hereditary lymphedema: *ADAMTS3, CCBE1, FAT4, FLT4, FOXC2, GATA2, GJA1, GJC2, KIF11, MDFIC, PIEZO1, PIK3CA, PTPN14, RASA1, SOX18,* and *VEGFC*. However, STRING database analysis also showed no interaction of the *GJA4* gene with genes in the rasopathy pathway, which can also be causative of increased fetal nuchal translucency, namely *BRAF, CBL, HRAS, KRAS, LZTR1, MAP2K1, MAP2K2, MAP3K8, MAPK1, MRAS, NF1, NRAS, PPPC1B, PTPN11, RAF1, RASA2, RIT1, RRAS, SHOC2, SOS2,* and *SPRED1*. Thus, we describe a novel gene-human fetal phenotype association.

## Introduction

Prenatal ultrasound is a standard and widely used method for checking for potential birth defects in a developing fetus. If an abnormality is found, further tests such as fetal karyotyping, chromosomal microarray (CMA), tests for maternal and fetal infections, fetal echocardiogram, or magnetic resonance imaging (MRI) are indicated. Nuchal translucency is the measurement of the fluid-filled space at the back of a fetus's neck during a first-trimester ultrasound. A normal amount of fluid is present in all babies, but a thicker accumulation can indicate an increased risk for chromosomal defects, cardiac defects, or other genetic conditions [1].

By checking the nuchal translucency, we can detect around 80% of fetuses with Down syndrome or other major chromosomal defects at a false positive rate (FPR) of 5% [2]. The nuchal fold (NF) thickness is a measurement performed on prenatal ultrasound in the second trimester, and is the distance from the outer edge of the occipital bone to the outer edge of the skin in the midline. Detection of fetuses with increased nuchal translucency in routine first-trimester ultrasound screening has been widely used as a sensitive indication for fetal chromosomal abnormalities and/or fetal structural anomalies, such as congenital heart disorders or neurodevelopmental anomalies detected in later gestations. Fetuses with increased nuchal translucency and structural malformations are frequently associated with genetic abnormalities and have poor prognoses. Most cases (over 80%) don't find a clear cause using the standard prenatal diagnostic tests currently available. This lack of a definitive answer makes genetic counseling and deciding on the best clinical management challenging for both families and healthcare providers [1-3]. 

Only a small fraction (0.8-5.3%) of fetuses with an isolated increase in nuchal translucency (without any major anomalies) are found to have a disease-causing chromosomal aneuploidy/microdeletion/microduplication. Some of these cases will also result in poor birth outcomes. Therefore, there is a need for a comprehensive test that can diagnose chromosomal as well as single-gene disorders [2-4]. 

In standard prenatal testing, quantitative fluorescent polymerase chain reaction is used to screen for two things: maternal cell contamination (maternal cells mixed up in the fetal sample) and chromosomal aneuploidies (for chromosomes 13, 18, 21, X, and Y). In addition, for the past 15 years, CMA has been the first test of choice to detect pathogenic chromosomal copy number variants. In around 20% of cases with nuchal translucency between 3 mm and 3.4 mm, CMA will detect pathogenic copy number variants (CNVs); however, it cannot detect point mutations or very small insertions/deletions [4]. Thanks to major advances in molecular technologies, especially next-generation sequencing, and the fact that its cost has dropped significantly over time, whole-exome sequencing (WES) is now commonly used for both scientific research and clinical medical applications [5]. Recent research indicates that WES is a promising diagnostic tool. It can provide a genetic diagnosis for 9.1-32% of fetuses found to have a structural anomaly. Specifically, in fetuses with an increased nuchal translucency (with or without structural defects), WES yields a diagnosis in 3.2-21% of cases. The important caveat is that most of these studies were conducted on groups of high-risk pregnancies after karyotype and CMA had already ruled out known causes. This practice was followed because WES is generally more expensive and less effective than CMA at reliably detecting CNVs in Western countries. [5]. However, in India, the cost of WES is similar to that of CMA, hence WES is increasingly becoming the first test of choice in USG detectable fetal anomalies.

The blood flow in a human fetus is a highly flexible and adaptable system throughout the pregnancy, mainly because it uses three special connections or "shortcuts" called physiological shunts: the ductus arteriosus, the foramen ovale, and the ductus venosus [6]. The ductus venosus is a vital fetal blood vessel that allows oxygenated blood from the placenta to bypass the liver and flow directly into the inferior vena cava (IVC), a major vein leading to the heart. Agenesis of ductus venosus (ADV) occurs when this vessel is absent or terminates abnormally, leading to a lack of the normal shunt between the umbilical vein and the IVC. ADV is associated with an increased risk of genetic abnormalities, cardiac and extracardiac malformations, and adverse perinatal outcomes, such as fetal growth restriction and stillbirth. In fetuses with an ADV, the blood returning from the placenta via the umbilical vein can take one of two main alternative paths. In extrahepatic drainage, the umbilical vein bypasses the liver entirely and shunts blood directly to a major systemic vein or heart chamber. This drainage can lead to the IVC/iliac vein/renal vein/right atrium or, less commonly, the left atrium or the coronary sinus. In intrahepatic drainage, the umbilical vein connects to the liver's circulatory system via the portal sinus and then flows into the hepatic sinusoids. Crucially, this route exists without forming the normal ductus venosus. ADV is associated with structural abnormalities in approximately 65% of cases and with chromosomal defects in 17% of cases. In around 20-60% of cases, it is associated with cardiac, extracardiac (most commonly single umbilical artery), and chromosomal anomalies, Noonan syndrome, agenesis of the portal vein in as many as half of the cases, and hydrops fetalis in 30-50% of the cases. The expected outcome, or prognosis, for a fetus with an ADV is heavily influenced by two main factors: where the umbilical blood drains and whether other birth defects are present. When the umbilical vein drains outside the liver, there is a high risk of complications, including congestive heart failure and the complete failure of the portal venous system to develop. When the blood drains through the liver, it's rarely associated with other malformations, leading to a much better outcome since the liver isn't bypassed. ADV is found as an isolated finding (meaning no other problems are detected) in only 35-59% of cases. However, for these isolated cases, the prognosis is excellent, with more than 80% of babies having a normal outcome [7].

In this report, we identify for the first time a homozygous nonsense variant in the *GJA4* gene c.97delC (transcript ID NM_002060.3) or p.Arg33Alafs*98 or chr1:g.34794309delC (GRCh38 format) in a human fetus with increased NF thickness and abnormal fetal ductus venosus termination, identified on WES.

## Case presentation

**Figure 1:**
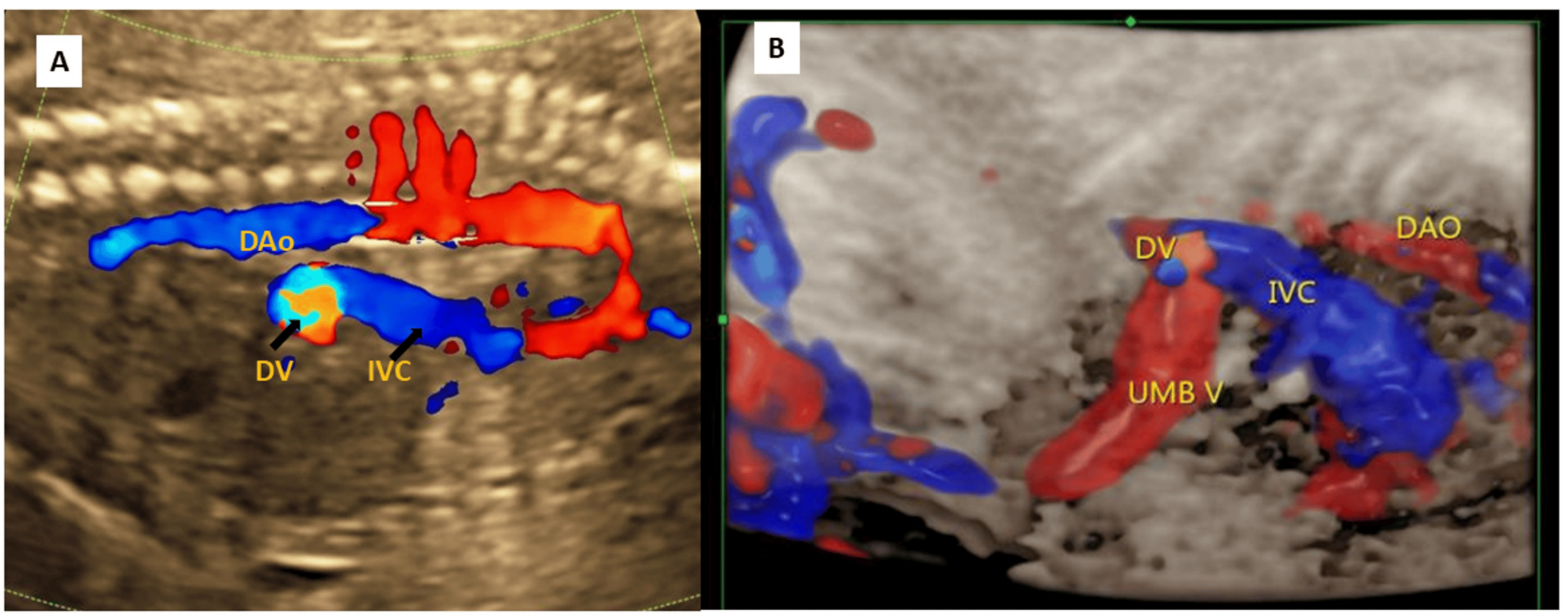
Doppler fetal ultrasound showing the abnormal termination of the ductus venosus. (A) Doppler ultrasound showing the sagittal plane of the fetal thorax and abdomen. There is abnormal termination of the DV (ductus venosus) in the infra-hepatic segment of the inferior vena cava (IVC). The descending aorta (DAo) is also seen. (B) Ultrasound of the fetus showing the sagittal plane of the fetal thorax and abdomen in cardiac STIC (spatio-temporal image correlation)-four-dimensional, showing the umbilical vein (UMB V) directly entering the infra hepatic segment of the IVC. The DV is seen immediately before the termination of the umbilical vein in the IVC. The descending aorta (DAO) is also seen.

A 21-year-old female patient, married in a non-consanguineous union, presented in her first pregnancy. Her first trimester scan around 12 weeks showed a nuchal translucency of 2.6 mm at a crown rump length of 58 mm (96th centile). The ductus venosus showed abnormal termination, opening in the infrahepatic part of the vena cava, the fetal oxygenated blood returning to the fetal heart, thus bypassing the liver (Figure 1).

**Figure 2:**
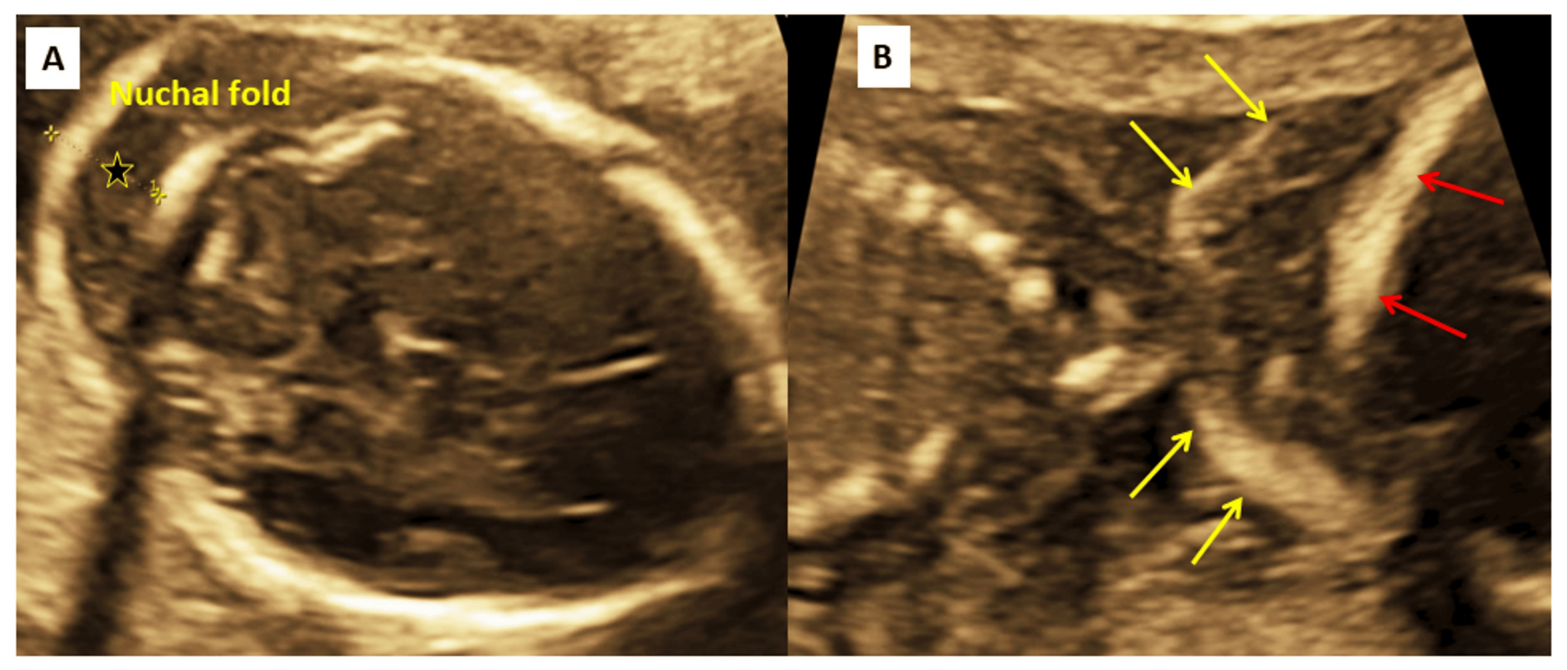
Ultrasound of the fetus showing increased nuchal translucency (A) Ultrasound of the fetus showing the axial image of the fetal cerebellar plane. The nuchal fold thickness (10 mm) is increased (shown by a star) (B) Ultrasound of the fetus showing the coronal image of the fetal neck from the back, showing increased deposition of fat and grossly increased skin thickness (yellow arrows – skin margin, red arrows – occipital bone).

The second-trimester ultrasound (performed around 20 weeks by the last ultrasound and 21 weeks one day by the last menstrual period) showed the fetal anthropometry as follows: biparietal diameter (BPD) 46 mm (52nd centile), occipito-frontal diameter (OFD) 61.9 mm (90th centile), BPD/OFD ratio 0.74 (9th centile), head circumference (HC) 170.6 mm (33rd centile), ventricular diameter was 6 mm (normal), trans-cerebellar diameter 18.8 mm (10th centile), abdominal circumference (AC) 135.1 mm (18th centile), femur length (FL) 30.5 mm (28th centile), FL/BPD ratio was 0.66 (20th centile), humerus 30.2 mm (58th centile), HC/AC ratio 1.26 (95th centile), estimated fetal weight 283 gms (17th centile). The NF was increased to 10 mm (normal is less than 6 mm). The pre-nasal edema and the skin thickness on the upper part of the back were also increased (Figure 2).

The following structures appeared normal: intracranial anatomy, face, heart, great vessels, thorax, abdomen, spine, extremities, and skeleton. There was abnormal termination of the ductus venosus opening in the infrahepatic part of the vena cava, thus bypassing the liver. The Doppler performed on the umbilical artery and the uterine arteries showed normal findings.

**Figure 3:**
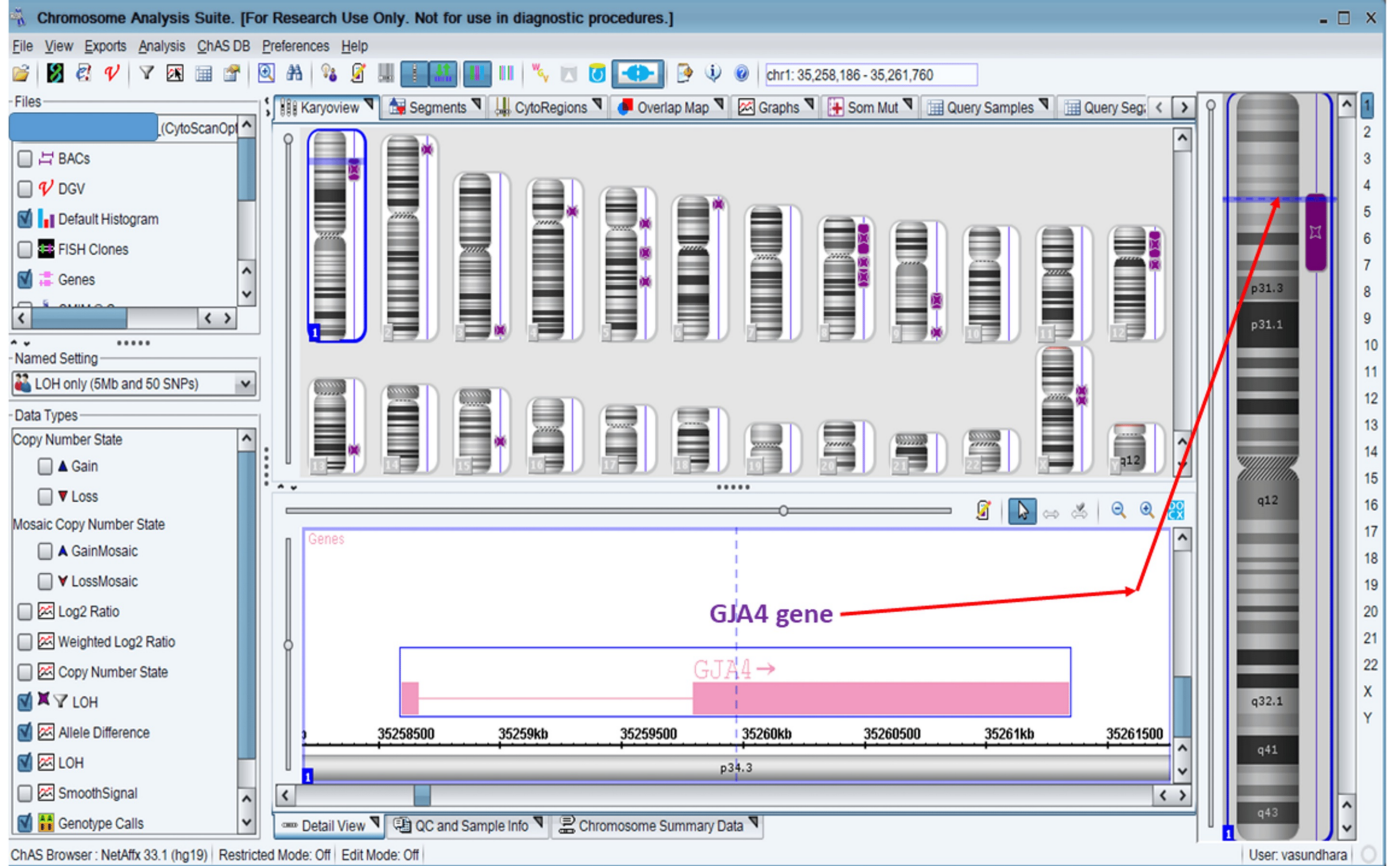
Virtual karyogram of the fetal chromosomal microarray Virtual karyogram of a chromosomal microarray performed on an Affymetrix 315K Optima chipset (Thermo Fisher Scientific Inc., Waltham, Massachusetts, United States) showing normal chromosomal number but a region of loss of heterozygosity (red arrow) (depicted by purple bands next to chromosome ideograms) on the p arm of chromosome 1, including the *GJA4 *gene

Amniocentesis was performed with patient consent. Chromosomal microarray performed on fetal DNA showed a normal chromosomal complement but a region of loss of heterozygosity on chromosome 1 (arr[hg19] 1p35.1p32.1(33,725,730-59,483,255) hmz), which included the *GJA4* gene (Figure 3).

**Figure 4:**
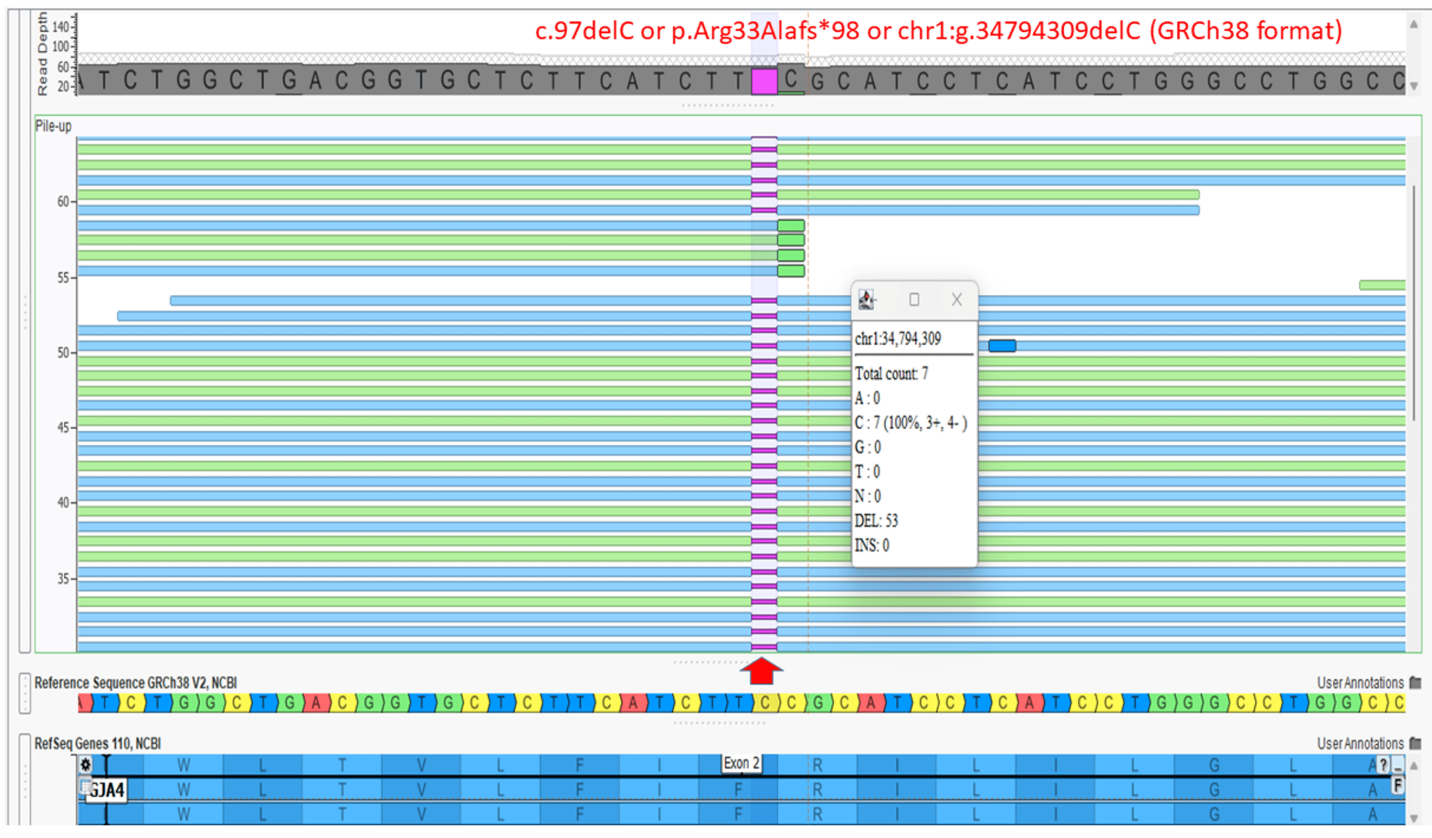
Screenshot of the Integrated Genomic Viewer after whole-exome sequencing Screenshot of the Integrated Genomic Viewer (VarSeq; Golden Helix Inc., Bozeman, Montana, United States) of the binary alignment map (BAM) file of the whole-exome sequence data for the affected fetus shows a homozygous variant in the *GJA4* gene, c.97delC (transcript ID NM_002060.3) or p.Arg33Alafs*98, or chr1:g.34794309delC (GRCh38 format). The position of the variant is shown by a red arrow.

WES was performed on the fetus using a commercial test (Twist exome panel 2.0; Twist Bioscience, South San Francisco, California, United States) that analyzes all 20,321 protein-coding genes and also includes coverage for 37 mitochondrial genes. The analysis looked for both single-nucleotide variants (changes in a single DNA base) and small insertions/deletions (InDels). In addition to these small changes, CNVs were detected from the same sequencing data using a validated software tool called ExomeDepth (version 1.1.10) [8]. This testing was conducted in a lab that meets high-quality standards, as it is accredited by the National Accreditation Board for Testing and Calibrating Laboratories (NABL) of India. The fetus was identified to be homozygous for a novel variant in the *GJA4* gene c.97delC (transcript ID NM_002060.3) or p.Arg33Alafs*98 or chr1:g.34794309delC (GRCh38 format) (Figure 4)

**Figure 5:**
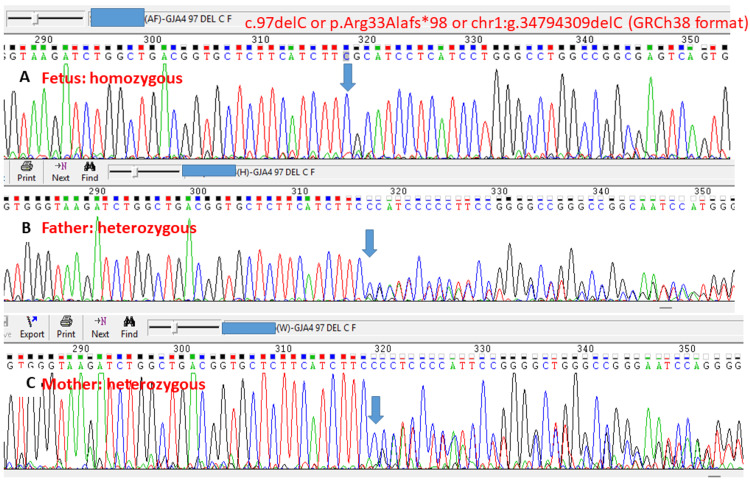
Results of Sanger sequencing analysis shows the variant in the fetus (A) in a homozygous state and in father (B) and mother (C) in heterozygous state Sequence chromatogram for the variant in the *GJA4* gene, c.97delC (transcript ID NM_002060.3) or p.Arg33Alafs*98, or chr1:g.34794309delC (GRCh38 format). The position of the variant is shown by blue arrow.

**Figure 6:**
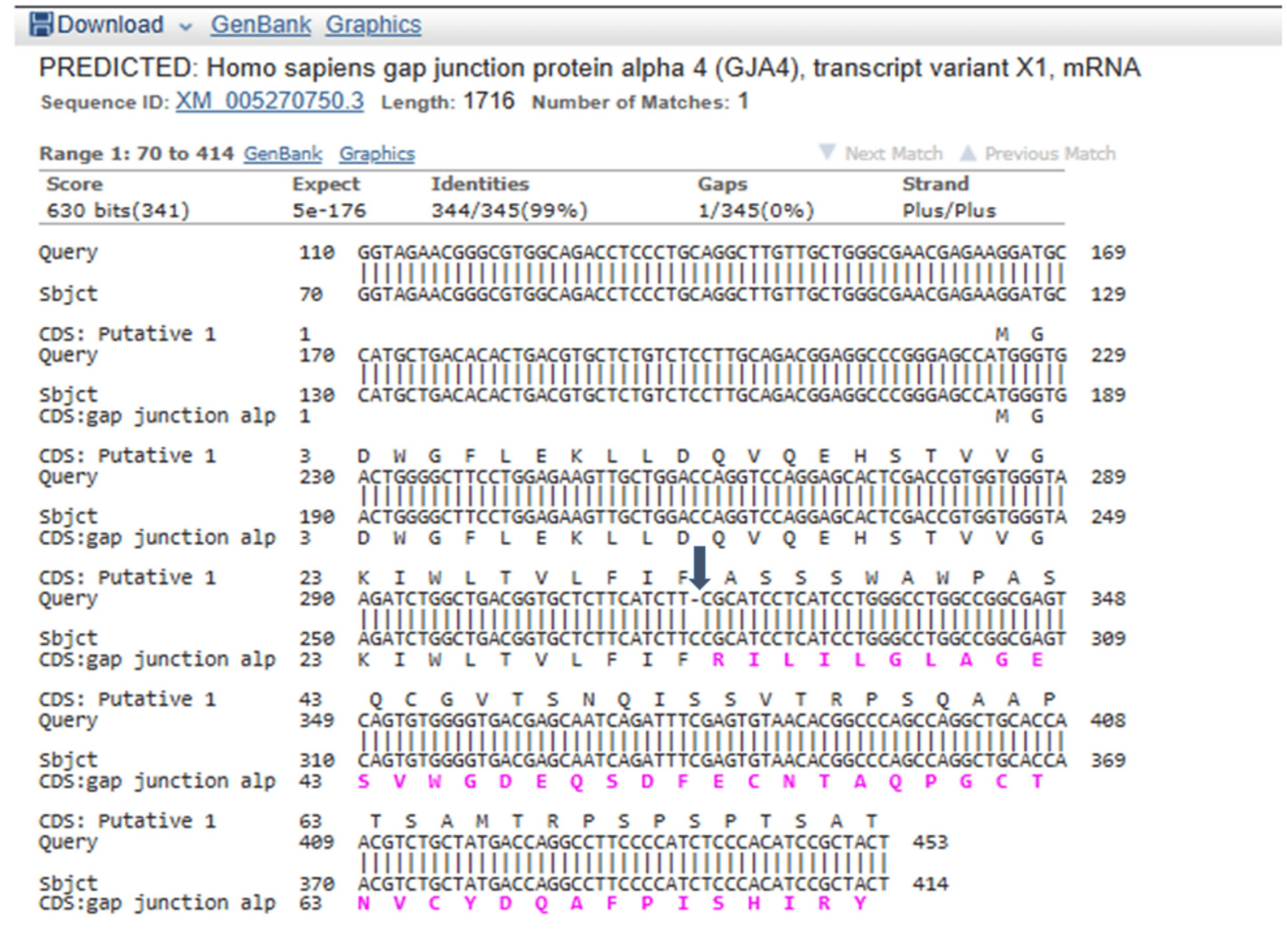
Basic Local Alignment Search Tool (BLAST) analysis of the sequence chromatogram BLAST analysis of the sequence chromatogram showing deletion of the nucleotide C (cytosine) at the c.97 position (transcript ID NM_002060.3) or chr1:g.34794309delC of the *GJA4* gene (GRCh38 format) leading to variant  p.Arg33Alafs*98.

This variant is present in the gnomAD database at a very low frequency (0.00003283). This variant is absent in the ClinVar public database (National Center for Biotechnology Information, National Institutes of Health, Bethesda, Maryland, United States). The variant is a variant of uncertain significance according to the American College of Medical Genetics pathogenicity criteria, satisfying PM2 (pathogenic moderate criteria 2: extremely low frequency in gnomAD database) [9]. This variant is present in a homozygous state in one apparently normal individual in gnomAD (https://gnomad.broadinstitute.org/variant/1-34794308-TC-T?dataset=gnomad_r4). However, this likely shows variable expressivity and incomplete penetrance of this variant, but does not preclude pathogenicity. For example, the known pathogenic variant in the *TBXAS1* gene c.1235G>A or p.Arg412Gln leading to Ghosal hematodiaphyseal dysplasia, is present in a homozygous state in gnomAD in one apparently normal individual, which shows variable expressivity and incomplete penetrance (https://gnomad.broadinstitute.org/variant/7-139715531-G-A?dataset=gnomad_r2_1) [10]. Exome copy number variant analysis did not reveal any significant chromosomal microdeletions/ microduplications. The asymptomatic couple was identified to be heterozygous for the same variant in the GJA4 gene by targeted Sanger sequencing of 700 nucleotides surrounding this region (Figures 5, 6).

Thereby, this variant showed an autosomal recessive pattern of inheritance. The couple was counselled about follow-up of the pregnancy; however, they declined to continue the pregnancy owing to the increasing fetal edema. The possibility of a recurrence rate of 25 % in every pregnancy due to autosomal recessive inheritance was counselled to the couple. However, since the genotype-phenotype relation is not established, definitive prenatal testing in this family, based on the *GJA4 *gene variant alone, was not advised.

Ethical considerations

Informed consent has been obtained from the patient's family for publication in an open-access journal. The study has been done in accordance with the principles of the Helsinki declaration (World Medical Association, 2013) [11]. The study received a formal waiver from the Institutional Ethics Committee of the Dr. D.Y. Patil Medical College, Hospital and Research Centre (approval number: I.E.S.C/W/63/2025).

## Discussion

In this report, we identify for the first time a homozygous nonsense variant in the *GJA4* gene c.97delC or p.Arg33Alafs*98, or chr1:g.34794309delC (GRCh38 format) in a human fetus with increased NF thickness and abnormal fetal ductus venosus termination, identified on whole-exome sequencing.

Biology of the *GJA4 *gene

The *GJA4* gene encodes connexin-37 (Cx37), a protein that forms gap junctions, allowing the passage of small molecules and ions between cells, and is expressed in various tissues, including the heart, kidney, and vascular system, playing roles in electrical coupling, vascular remodeling, and angiogenesis. Reed et al. (1993) used polymerase chain reaction (PCR) amplification and cDNA library screening to clone DNA encoding the connexin-37 gap junction protein. The derived polypeptide contained 333 amino acids, with a predicted molecular mass of about 37 kDa [12]. Angelillo-Scherrer et al. (2011) found that Cx37 -/- mice had shortened bleeding time after injury compared to controls, with more rapid and extensive formation of thrombus due to increased platelet aggregation. Similarly, blocking CX37 channels increased aggregation of human platelets, confirming the involvement of CX37 channels in limiting platelet aggregation. Neurobiotin transfer assay showed that Cx37 formed functional gap junction channels during platelet aggregation. Expression of CX37 c.1019C-T, a single nucleotide polymorphism resulting in a P319S substitution in the regulatory C terminus of the CX37 protein, in communication-deficient HeLa cells led to larger channel permeability [13].

The Jackson laboratory describes in detail a mouse *Gja4* gene knock-out model. These mice have a mutation where the *Gja4* gene's coding section is largely replaced by a PGK-NEO cassette. As a result, in the mice with two copies of this mutation (homozygotes), no gene product (like mRNA or protein) could be detected in various tissues, like the heart, oocytes, aorta, kidney, and platelets. Homozygous females are viable but infertile because their ovulation is absent; their oocyte and ovarian follicle development stop. Homozygous males and mice with only one copy of the mutation (heterozygous females) are both viable and fertile. Homozygotes have an increased number of collateral blood vessels. They recover faster from an experimental hind limb injury involving ischemia, showing enhanced remodeling and angiogenesis compared to controls. Vasoconstriction in the arterioles of the cremaster muscle is reduced. Bleeding time is reduced. They show more rapid thrombus formation and increased platelet aggregation. As embryos (at day 13.5), they have enlarged jugular lymph sacs. At later stages (embryos and adults), they have fewer lymphatic valves, including those in the thoracic duct, leading to lymphatic dysfunction. They lack venous valves in their peripheral veins [14].

Another study by Wong et al showed that the *Gja4* gene protects against atherosclerosis in mice. Mice that are genetically engineered to lack both the Cx37 gene (*Gja4* −/− ) and a gene that prevents high cholesterol (*Apoe* −/− ) develop more severe atherosclerosis of the arteries (aortic lesions) than mice that only lack the *Apoe* gene (*Gja4* +/+ *Apoe* −/− ). The researchers found that Cx37 protects arteries by specifically acting in immune cells (monocytes and macrophages), not in the cells that line the blood vessels (endothelium). When the Cx37 gene is deleted, there is enhanced recruitment of monocytes and macrophages to the artery walls, which is the first step in forming plaque. The anti-adhesive mechanism: the Cx37 protein acts as a hemichannel on the surface of these immune cells. A hemichannel is essentially a small pore or gate. This Cx37 hemichannel is responsible for releasing ATP into the space outside the cell. The release of ATP is what inhibits the ability of the monocytes and macrophages to stick to the artery wall. Therefore, when Cx37 is missing, less ATP is released, the cells become more sticky, and more plaque forms [15].

Association of the *GJA4* gene and human disease

Ugwu et al. identified a recurrent *GJA4* c.121G>T (p.Gly41Cys) somatic mutation (in hepatic lesions) in 12 of 14 unrelated individuals with hepatic hemangiomas and in cutaneous venous malformations in three unrelated individuals. The p.Gly41Cys variant is located in the first transmembrane domain at a specific site that has remained virtually conserved among vertebrates. The variant altered the cell shape and activated serum glucocorticoid-regulated kinase 1 (SGK1) (which controls cell growth and death) via a non-canonical pathway. Therapy with spironolactone, a known inhibitor of angiogenesis, decreased SGK1 activation by the variant and reversed the morphological changes induced by the variant [16]. 

Hongo and colleagues discovered that the same somatic genetic change, c.121G>T (p.Gly41Cys) in 25 out of 26 cases of orbital cavernous venous malformation (OCVM). OCVM is a non-hereditary (sporadic) blood vessel abnormality of unknown origin, defined by its characteristic abnormally widened and dilated blood channels. An electrical analysis technique called whole-cell voltage clamp was performed on frog egg cells (Xenopus oocytes). This testing showed that the *GJA4* c.121G>T (p.Gly41Cys) variant is a gain-of-function mutation. This means the resulting protein structure, a hemichannel, is abnormally active, or hyperactive, allowing it to function much more vigorously than normal. When the mutant protein was excessively produced in human umbilical vein endothelial cells, it caused the cells to lose their cellular integrity. This loss of structure was corrected when the cells were treated with carbenoxolone, a drug that blocks both gap junctions and hemichannels. This evidence suggests two important things: the *GJA4* c.121G>T (p.Gly41Cys) change is a likely "driver" mutation for venous malformations and that the resulting hyperactive hemichannel plays a key role in the development of this specific vascular defect [17]. 

Thus, a loss-of-function variant, as in our case, can be expected to have a converse effect, leading to ductus venosus absence due to abnormal endothelial function. The cause of edema in the skin in our case could also be due to abnormal lymphatic drainage.

**Figure 7:**
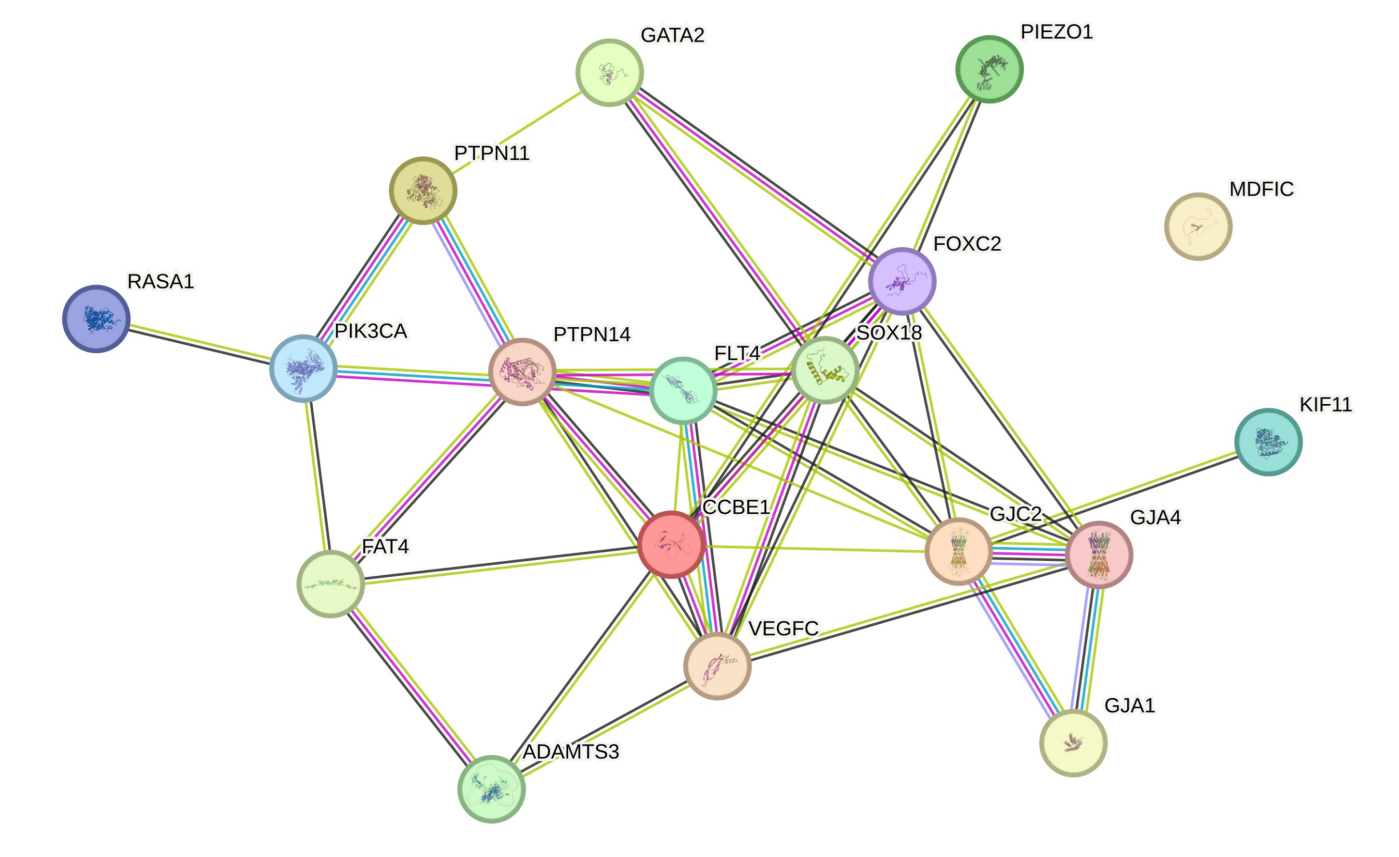
STRING database analysis of the GJA4 gene with genes involved in causation of hereditary lymphedema A STRING database analysis of genes showed close interactions between the *GJA4* gene and other genes involved in the causation of hereditary lymphedema: *ADAMTS3, CCBE1, FAT4, FLT4, FOXC2, GATA2, GJA1, GJC2, KIF11, MDFIC, PIEZO1, PIK3CA, PTPN14, RASA1, SOX18, *and* VEGFC.* STRING: Search Tool for Retrieval of Interacting Genes/Proteins Image Credit: Authors; created online at https://string-db.org/

We also performed a protein-protein network analysis using the STRING (Search Tool for Retrieval of Interacting Genes/Proteins) database [18], which showed close interactions between the* GJA4* gene and other genes involved in the causation of hereditary lymphedema, *ADAMTS3, CCBE1, FAT4, FLT4, FOXC2, GATA2, GJA1, GJC2, KIF11, MDFIC, PIEZO1, PIK3CA, PTPN14, RASA1, SOX18,* and VEGFC (Figure 7).

**Figure 8:**
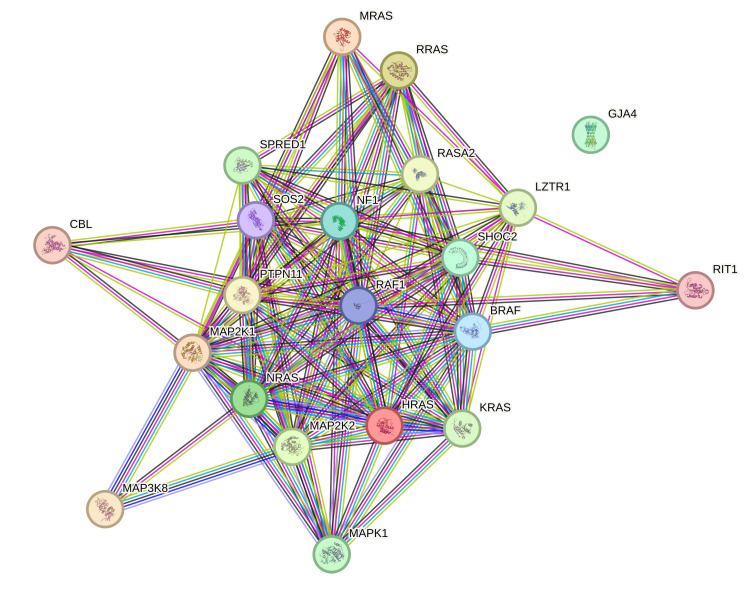
STRING database analysis of the GJA4 gene with genes in the rasopathy pathway A STRING database analysis of genes showed no interaction of the GJA4 gene with genes in the rasopathy pathway, namely *BRAF, CBL, HRAS, KRAS, LZTR1, MAP2K1, MAP2K2, MAP3K8, MAPK1, MRAS, NF1, NRAS, PPPC1B, PTPN11, RAF1, RASA2, RIT1, RRAS, SHOC2, SOS2, *and* SPRED1.* STRING: Search Tool for Retrieval of Interacting Genes/Proteins Image Credit: Authors; created online at https://string-db.org/

However, STRING database analysis also showed no interaction of the *GJA4* gene with genes in the rasopathy pathway, which can also be causative of increased fetal nuchal translucency, namely *BRAF, CBL, HRAS, KRAS, LZTR1, MAP2K1, MAP2K2, MAP3K8, MAPK1, MRAS, NF1, NRAS, PPPC1B, PTPN11, RAF1, RASA2, RIT1, RRAS, SHOC2, SOS2, *and *SPRED1* (Figure 8). 

Thus, we describe a novel gene-human fetal phenotype association. However, the variant remains of uncertain significance pending further research on loss-of-function type variants in the *GJA4* gene.

## Conclusions

In this report, we identify for the first time a homozygous nonsense variant in the *GJA4* gene, c.97delC or p.Arg33Alafs*98 or chr1:g.34794309delC (GRCh38 format) in a human fetus with increased NF thickness and abnormal fetal ductus venosus termination. The asymptomatic parents were carriers of the same variant, suggesting an autosomal recessive inheritance pattern. This variant is predicted to be a loss-of-function variant due to the absence of protein, due to nonsense-mediated decay of the mRNA. The association of a variant in the *GJA4* gene with human fetal disease has not been previously reported, as per our search in the available medical literature.

We postulate that loss of function of the *GJA4* gene produces absence of ductus venosus, a converse effect to gain of function mutation (generation of vascular malformations in skin and liver, and orbit), and fetal nuchal edema due to lymphatic dysfunction. Further research is required to confirm or negate this association. A limitation of this study is the non-availability of functional analysis of this specific variant using RNA or protein studies or cellular or animal modelling. This report will aid in the diagnosis and genetic counseling of families with variants in the *GJA4* gene. 
